# *AtGRP3* Is Implicated in Root Size and Aluminum Response Pathways in *Arabidopsis*

**DOI:** 10.1371/journal.pone.0150583

**Published:** 2016-03-03

**Authors:** Amanda Mangeon, Renan Pardal, Adriana Dias Menezes-Salgueiro, Guilherme Leitão Duarte, Ricardo de Seixas, Fernanda P. Cruz, Vanessa Cardeal, Claudia Magioli, Felipe Klein Ricachenevsky, Rogério Margis, Gilberto Sachetto-Martins

**Affiliations:** 1 Laboratório de Genômica Funcional e Transdução de Sinal, Departamento de Genética, Universidade Federal do Rio de Janeiro, Rio de Janeiro, 21941–617, Brazil; 2 Programa de Pós-Graduação em Botânica (PPGBot), Universidade Federal do Rio Grande do Sul, Porto Alegre, RS, 91501–970, Brazil; 3 Departamento de Biologia, Universidade Federal de Santa Maria, Santa Maria, RS, 97105–900, Brazil; 4 Centro de Biotecnologia e Departamento de Biofísica da Universidade Federal do Rio Grande do Sul, Porto Alegre, RS, 91501–970, Brazil; Instituto de Biología Molecular y Celular de Plantas, SPAIN

## Abstract

AtGRP3 is a glycine-rich protein (GRP) from *Arabidopsis thaliana* shown to interact with the receptor-like kinase AtWAK1 in yeast, *in vitro* and *in planta*. In this work, phenotypic analyses using transgenic plants were performed in order to better characterize this GRP. Plants of two independent knockout alleles of *AtGRP3* develop longer roots suggesting its involvement in root size determination. Confocal microscopy analysis showed an abnormal cell division and elongation in *grp3-1* knockout mutants. Moreover, we also show that *grp3-1* exhibits an enhanced Aluminum (Al) tolerance, a feature also described in *AtWAK1* overexpressing plants. Together, these results implicate *AtGRP3* function root size determination during development and in Al stress.

## Introduction

The plant glycine-rich proteins (GRPs) superfamily is characterized by the presence of variable semi-repetitive glycine-rich motifs. Based on these variations, this superfamily has been further divided into five distinct classes. This classification does not consider these protein functions due to the fact that only recently the first functional characterization studies were performed, elucidating some plant GRP activities [1].

Plant GRPs have been of scientific interest due to their tissue-specific, developmentally and/or stress modulated expression patterns (reviewed in [2]). GRPs have been identified in various plant species, and over 150 *GRP* genes were found in the transcriptome or whole-genome analysis of sugarcane, *Eucalyptus*, *Arabidopsis* and rice [3, 4]; V. Galvão, V. Cardeal and G. Sachetto-Martins, personal communication).

Functional characterization approaches have been conducted in order to study plant GRP function (reviewed in [1]). Most of these studies focused on *Arabidopsis* GRPs and have implicated plant GRPs in pollen hydration and competition [5], flowering [6]; [7], plant defense [8], RNA splicing [9], cell elongation [10], pri-miRNA processing [11] and various responses including cold and osmotic stress [12–20].

The *AtGRP3* gene (At2g05520) was first isolated as a cDNA clone from *Arabidopsis* and Northern blot analysis indicated strong expression of this gene in leaves and inflorescence axis. The protein sequence contains a putative signal peptide, followed by a glycine-rich region with GGXXXGG motif and a cysteine-rich C-terminus [21]. This structure classifies AtGRP3 as a Class II GRP [1]. The cysteine-rich domain is necessary for the interaction of AtGRP3 with the extracellular domain of the wall associated kinase AtWAK1 [22]. AtWAK1 (At1g21250) is a receptor-like kinase (RLK) containing an extracellular, a transmembrane and a cytoplasmic kinase domain [23]. This gene is expressed throughout plant development and is induced by an analog of salicylic acid [24]. Sub-cellular localization experiments using GFP fusion indicated that AtWAK1 is initially localized to endomembrane system and then transported to the cell surface where it is co-localizes with pectin as shown by protoplast experiments [25, 26]. Domain swap studies showed that binding of oligogalacturonides to the extracellular domain of AtWAK1 triggers activation of the kinase domain eliciting defense responses against fungi and bacteria. Accordingly, plants overexpressing AtWAK1 are more resistant to the fungus *Botrytis cinerea* [27]. These plants also display an enhanced Al tolerance suggesting a role for *AtWAK1* in Al signaling pathway [28].

AtGRP3/AtWAK1 binding has been shown not only through yeast two-hybrid experiments, but has also been confirmed *in vitro* and *in planta*. In addition, a protein complex involving AtGRP3/AtWAK1 and the kinase-associated protein phosphatase (KAPP) is formed *in planta*. AtGRP3 expression is induced by salicylic acid resulting in a positive feedback that stimulates further its expression as well as the expression of *AtWAK1* and *PR-1* in protoplasts, suggesting a role for *AtGRP3* in plant defense and signaling [22].

Here, in order to elucidate the functional role of *AtGRP3* throughout plant development and its possible involvement in AtWAK1-mediated Al signaling, knockout plants were characterized. Our results propose the participation of *AtGRP3* in determining root size. These results are confirmed by confocal microscopy analysis, which indicates an abnormal cell division and cell elongation in *grp3-1* knockout mutants. Finally, *grp3-1* knockout plants presented enhanced Al tolerance, suggesting that AtGRP3 and AtWAK1 function in the same signaling pathway.

## Material and Methods

### Plant material

Growth conditions, root growth analysis were performed according to Mangeon and collaborators [10]. Root growth experiments in Al were performed according to Sivaguru and collaborators [28]. The growth measurements were performed 10 days after seedling transfer to plates containing Aluminum chloride hexahydrate, 99% (hereafter, Al).

### T-DNA lines

The *grp3-1* T-DNA mutant, SALK_084685, was isolated from the Salk Institute Genomic Analysis Laboratory collection [29]. Homozygous mutants were isolated by PCR-based genotyping using gene specific PCR primers G3 LP (5’CCAACGCTTTGAAAAAGTTAAA3´) and G3 RP (5´tgaattcactgtggctgtccaaa3´) together with LBa1 (5’TGGTTCACGTAGTGGGCCATCG3’). A second T-DNA insertion line, *grp3-2* T-DNA mutant, SALK_012941c, was isolated from the Salk Institute Genomic Analysis Laboratory collection as an homozygous line.

### Real-time quantitative PCR (RT-qPCR)

The RT-qPCR experiments were carried out on cDNAs synthesized from total RNA extracted from 5 days-old seedlings using Trizol (Thermo-Fischer) according to the manufacturer’s instructions. One μg of total RNA was pre-digested with RQ1 RNase-free DNase (Promega) following manufacturer’s protocol and was used to synthesize cDNA using Superscript III (Thermo-Fischer) according the manufacturer’s instructions. Real-time quantitative PCR reactions were performed using SYBR Select Master Mix (Thermo-Fischer) in standard conditions. *TIP41* (At4g34270) and *FDH* (At5g43940) were used as reference genes. A list of primers and concentrations used is presented in S1 Table. Reactions were performed in an Applied Biosystems 7500 Fast real-time PCR system and results were analyzed according to LinReg PCR (HFRC) and qBase (Biogazelle).

For the expression analysis, five pools containing 10 plants each were used in the experiments. The plotted data is an arithmetic mean of the three pools presenting the observed trend, excluding the outliers. For each sample, three technical replicates were performed.

### Confocal microscopy analyses

For confocal visualization of root cells, plants were stained with propidium iodide according to Truernit and collaborators [30]. Analyses were performed in a Leica TCS SPE instrument using settings for propidium iodide according to the manufacturer (Leica Microsystems). Measurements were performed using ImageJ software (NIH).

For root diameter and number of cell rows analysis, eight plants of each background were used. For root length analysis, one hundred cells for each background were measured at the root hair zone.

### Statistical analysis

The phenotypic parameters were analyzed according to the Student test (*t* test) for comparison between arithmetic means of samples in which the variances are different. The probability of random events is 95% and only values of *P*<0.05 were considered.

## Results

### Phenotypic analyses indicate that *AtGRP3* is involved in determining root size

**Fig 1:**
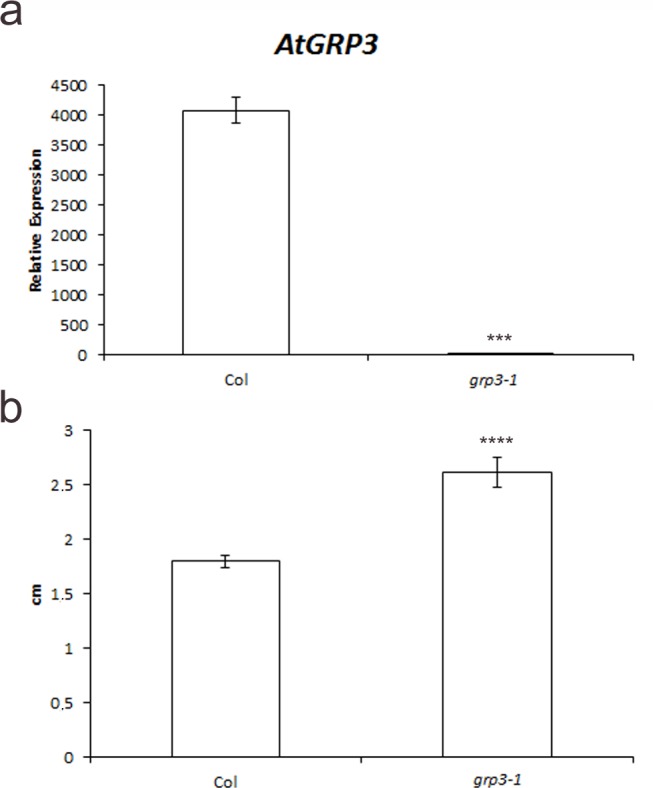
*grp3-1* loss-of-function mutant analysis. **a** Relative expression of *AtGRP3* transcripts analyzed through real-time quantitative PCR of Col and *grp3-1* mutant. **b** Summarized data for root length measurements of 2-week-old plants. *Error bars* indicate standard error. *** indicates p≤ 0.005 and **** indicates p≤ 0.001.

In order to characterize the functional role of *AtGRP3*, a loss-of-function line was obtained. This T-DNA line from the Salk Collection presenting insertion in the 5’UTR was genotyped and homozygous lines were selected. Quantitative real-time PCR analysis demonstrated that this line, named *grp3-1*, corresponds to an effective knockout without detectable levels of transcripts (Fig 1A).

Phenotypical analyses of *grp3-1* knockout plants were carried out throughout plant development and we observed that *grp3-1* knockout plants presented a 45% increase in root length compared to Col, used as controls (Fig 1B).

In order to confirm if the observed phenotype was due to loss of *AtGRP3* function, a second T-DNA line (named *grp3-2*) was also analyzed. Quantitative real-time PCR analysis indicates that this line is also a knockout allele (S1A Fig). Phenotypical analyses of *grp3-2* were carried out in order to confirm the increase in root length observed in the other *AtGPR3* mutant allele. Indeed, *grp3-2* also present longer roots compared to Col (S1B Fig) corroborating the hypothesis that *AtGRP3* is involved in determining root size.

### Cell elongation and division markers are induced in *grp3-1* knockout plants

The size of plant organs are controlled by two main processes: cell elongation and cell division [31–34]. In order to verify the cause for the enhanced root size observed in *grp3-1* plants, the expression of genes known to be involved in these two processes was assessed.

**Fig 2:**
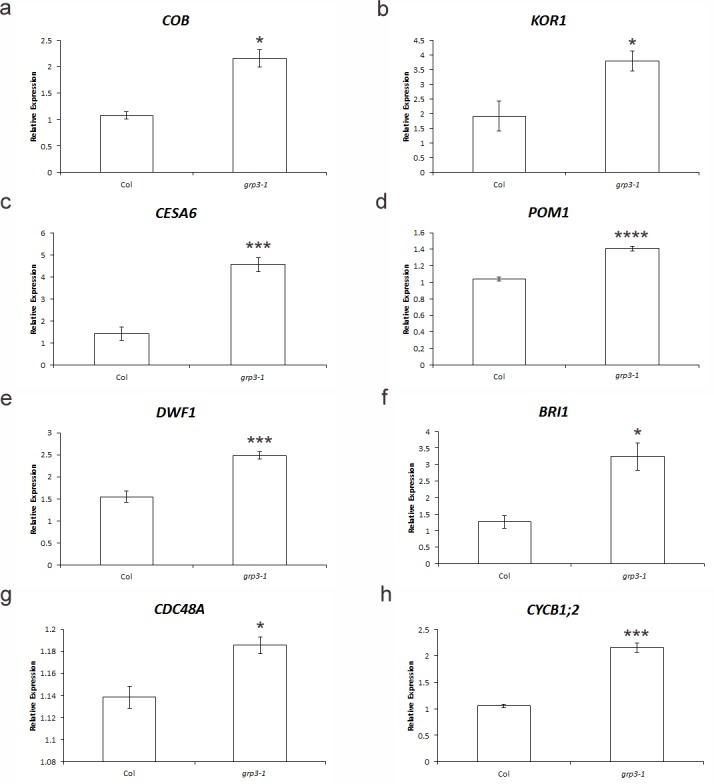
Relative expression of cell elongation and/or division molecular markers in Col and *grp3-1*. Quantitative real time PCR for **a** *COB* (At5g60920). **b** *KOR1* (At5g49720). **c** *CESA6* (At5g64740). **d** *POM1* (At1g05850). **e** *DWF1* (At3g19820). **f** *BRI1* (At4g39400). **g** *CDC48* (At3g09840). **h** *CYCB1;2* (At5g06150). *Error bars* indicate standard error. * indicates p≤ 0.05, *** indicates p≤ 0.005 and **** indicates p≤ 0.001.

First, genes involved in cell wall biosynthesis [35–38] and modification [39, 40] were tested. In *grp3-1* plants, a 2-fold induction in the expression of both the cellulose biosynthesis regulator gene *COBRA* (*COB*, At5g60920) and the endo-1,4-β-glucanase gene *KORRIGAN1* (*KOR1*, At5g49720) was observed (Fig 2A and 2B). Furthermore, a 220% increase in the cellulose synthase gene (*CESA6*, At5g64740) expression was also seen in *grp3-1* compared to wild-type (Fig 2C). For the chitinase-like gene *POM1* (At1g05850), an increase of 35% was detected in *grp3-1* (Fig 2D).

Since the phytohormone brassinosteroid is involved in cell elongation processes among other functions [41], genes involved in brassinosteroid biosynthesis [42] and signaling [43, 44] such as the brassinosteroid receptor gene *BRI1* (At4g39400) and the brassinosteroid biosynthesis gene *DWF1* (At3g19820) were also analyzed. A 60% and 157% increase over Col expression were detected for *DWF1* and *BRI1* in *grp3-1*, respectively (Fig 2E and 2F).

Genes involved in cell division [45] and cell cycle [46] were also tested. The cell division cycle gene *CDC48A* (At3g09840) presented a modest, but significant induction (3%) (Fig 2G) while the mitotic cyclin *CYCB1;2* (At5g06150) had a 2-fold induction (Fig 2H).

### Microscopy analysis reveals enhanced cell elongation and abnormal cell division in *grp3-1* roots

**Fig 3:**
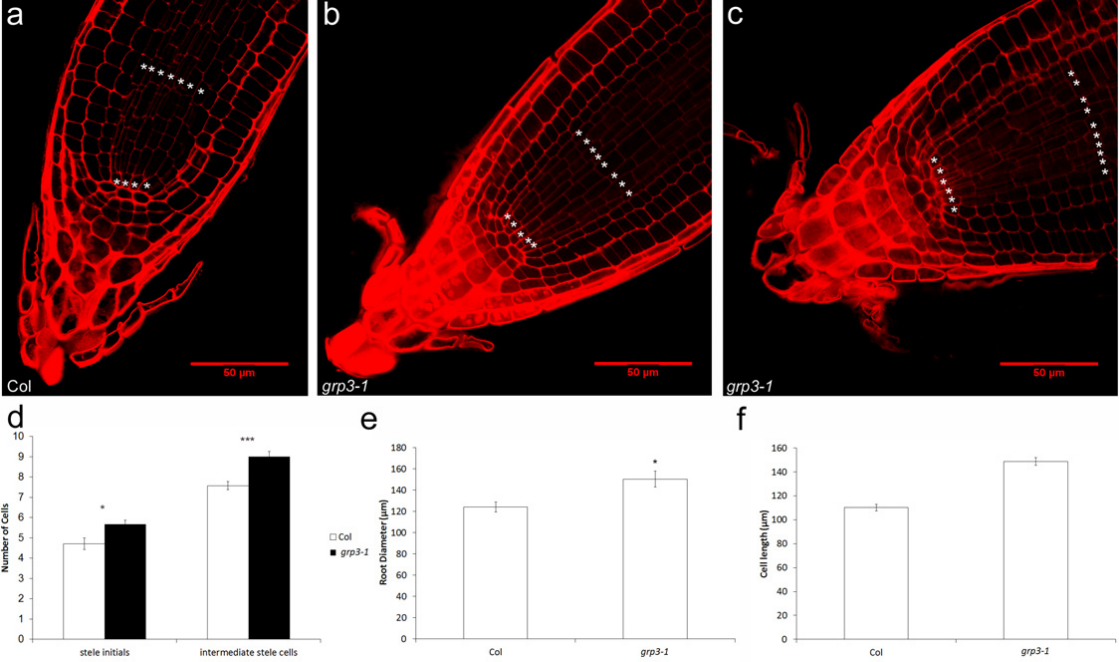
Confocal analysis of root cells. a-c Division pattern of stele cells. * labels stele rows. a Col wild-type. **b** *grp3-1* individual presenting normal division pattern. **c** *grp3-1* individual presenting abnormal division pattern. **d** Counting of number of stele cell rows. **e** Root diameter measurements. **f** Cell length measurements. *Error bars* indicate standard error. * indicates p≤ 0.05, ** indicates p≤ 0.01, *** indicates p≤ 0.005 and **** indicates p≤ 0.001.

In order to verify if cell division and elongation could be accounted for the enlarged root size phenotype seen in *grp3-1* mutants, confocal microscopy analysis was conducted. For that matter, root cells were measured and counted. The first noticeable difference was in the division pattern of stele cells in *grp3-1* compared to Col plants. While a small proportion of *grp3-1* individuals presented a pattern of division similar to Col (Fig 3A and 3B), over 70% of the individuals presented disorganized stele cell rows (Fig 3C). It is important to note that, even when the pattern of division was normal, all *grp3-1* plants presented extra rows of stele cells. On average, *grp3-1* plants presents two extra rows of stele cells compared to Col (Fig 3D). This increase is reflected in a 20% increase of the root diameter of *grp3-1* plants (Fig 3E).

In order to verify if cell elongation is also disturbed in *grp3-1* mutants, root cell length in the maturation zone was measured. Root cells in g*rp3-1* plants were 35% longer than Col root cells (Fig 3F), indicating that cell elongation is indeed contributing for the increase in root size. These observations corroborate the data shown above of higher expression levels of several cell elongation molecular markers in the *grp3-1* mutant.

### *grp3-1* presents an increased tolerance to Al

In a previous work, Sivaguru and collaborators [28] have reported that plants overexpressing AtWAK1 presented an increased tolerance to Al. Since AtGRP3 is capable of binding to AtWAK1 extracellular domain [22], we investigated if AtGRP3 was also involved in Al signaling by testing *grp3-1* plants for Al tolerance.

**Fig 4:**
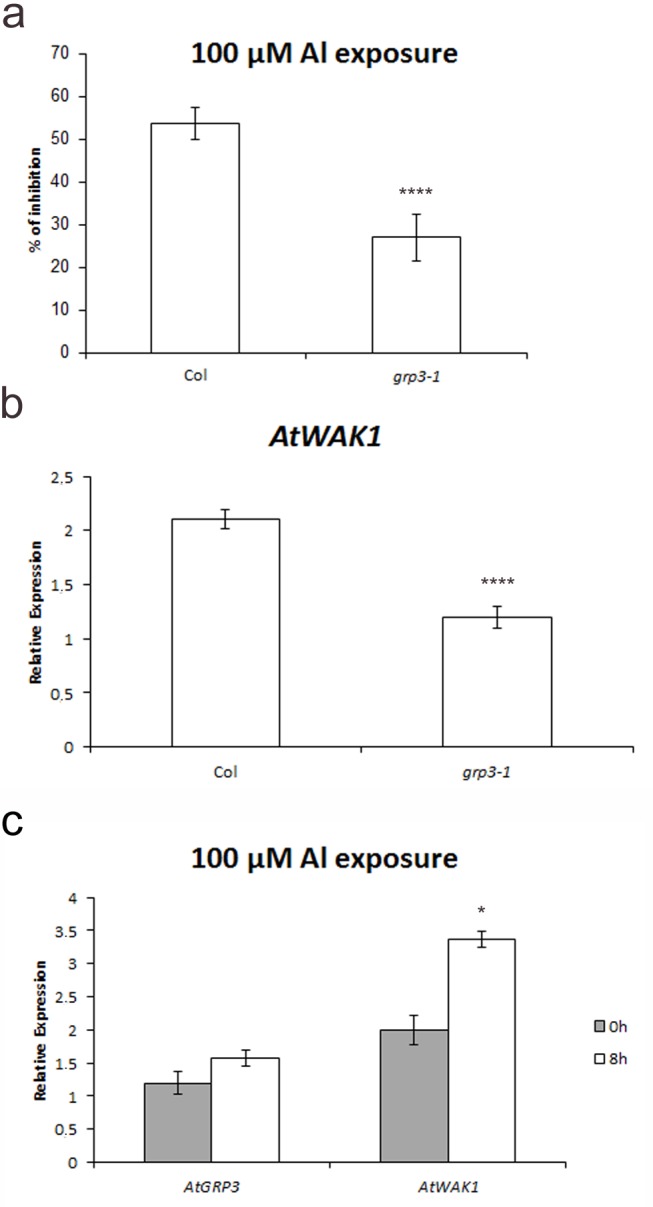
Analyses regarding implication of *AtGRP3* in Al signaling in Col wild-type and *grp3-1* knockout plants. **a** Reduction in root growth resulting from Al exposure. *Error bars* indicate standard error. **b** Relative expression of *AtWAK1* transcripts analyzed through real-time quantitative PCR of Col and *grp3-1* mutant. *Error bars* indicate standard error. *** indicates p≤ 0.005 and **** indicates p≤ 0.001. **c** Quantitative real time PCR for *AtGRP3* and *AtWAK1* in Col plants submitted to Al for 0h and 8h. *Error bars* indicate standard error. * indicates p≤ 0.05 and **** indicates p≤ 0.001.

Col plants submitted to Al presented an inhibition in root growth of 54%, while in *grp3-1* plants this inhibition was reduced to 27% (Fig 4A). This data suggests, therefore, that as observed for plants overexpressing AtWAK1, *grp3-1* knockout plants also present an increased tolerance to Al.

Park and collaborators [22] have shown that addition of AtGRP3 to protoplasts led to *AtWAK1* expression induction. In order to check if AtGRP3 is involved in *AtWAK1* endogenous expression, the levels of *AtWAK1* were analyzed in the *grp3-1* mutant. In fact, a 43% reduction of *AtWAK1* expression levels was observed in the *grp3-1* mutant compared to Col wild-type (Fig 4B).

Sivaguru and collaborators [28] have shown that *AtWAK1* expression levels were induced in the presence of Al. In order to verify if *AtGRP3* is also modulated by Al, Col plants were submitted to 8h of 100μM Al and the levels of *AtGRP3* were checked. Differently from *AtWAK1*, *AtGRP3* was not significantly modulated by Al (Fig 4C).

## Discussion

The analysis of two mutant alleles hints for a possible role of *AtGRP3* in determining root size. In plants, this control is regulated by two major events–cell division [33, 34] and cell elongation [31, 32]. A *GRP* gene from a different class–*AtGRP5* –presented organ size phenotypes that were caused mainly by altering cell elongation [10]. We found a similar phenotype of altered cell length in *grp3-1* knockout roots (Fig 3F) indicating that *AtGRP3* is another *GRP* gene involved in regulating cell elongation processes. Functional analyses though, suggest that they have opposing roles in cell elongation, since *AtGRP5* is a promoter [10] while *AtGRP3* works as a repressor of cell elongation.

Corroborating AtGRP3/AtWAK1 interaction previously reported [22] and the possible role of *AtGRP3* in root size determination, plants overexpressing *AtWAK1* also present shorter roots compared to wild-type [28]. Interestingly, the levels of *AtWAK1* are reduced in *grp3-1* mutant (Fig 4B) which displays longer roots. This suggests that both AtGRP3 and AtWAK1 work as repressors of root growth.

Kohorn [47] has proposed a model in which WAKs, GRPs and pectin together regulate cell expansion. Years later, corroborating Kohorn’s model, Decreux and Messiaen [48] have demonstrated that AtWAK1 binds pectin in vitro. Our results are in agreement with this model since *grp3-1* knockout plants present increased root cell length.

The most prominent phenotype of *grp3-1* mutant is its root length (Fig 1B). The analysis of expression levels of genes known to be involved in cell wall deposition (*COB*, *KOR1*, *CESA6*), cell wall modification (*POM1*) and brassinosteroid signaling (*BRI1*) has shown to be upregulated in *grp3-1* knockouts (Fig 2) which presents longer roots (Fig 1B). Agreeing with these data, null mutants for all those genes present shorter roots [35, 36, 39, 49].

Besides cell elongation, division also can be accounted for organ size [31–34]. In order to analyze if cell division markers are deregulated in the *grp3-1* knockout mutant, the expression of several cell cycle-related genes was assigned (Fig 2, S1 Table). *CYCB1;2* and *CDC48A* expression were in fact up-regulated in the *grp3-1* mutant (Fig 2G and 2H). Corroborating these data, confocal microscopy analysis has shown that *grp3-1* mutant present more stele cell rows (Fig 3A–3D). Interestingly, the analysis of a *CDC48A* mutant—a gene upregulated in the *grp3*-1 background—revealed a root tip free of stele cells [45].

Plants overexpressing the RLK AtWAK1 presented increased Al tolerance [28]. Since AtGRP3 binds to the extracellular domain of this protein [22], the idea that this signaling was also dependent of AtGRP3 is very tempting. It is expected that AtWAK1 overexpression plants contain an excess of AtWAK1 free of AtGRP3. With that idea in mind, *grp3-1* plants, in which AtWAK1 free of AtGRP3 is also present, were tested for Al tolerance. In fact, *grp3-1* knockout plants also displayed increased Al tolerance (Fig 4A). One hypothesis is that, in the presence of Al, AtGRP3 binding to AtWAK1 leads to physiological and morphological responses that result in root growth inhibition. Therefore, in the event of accumulation of AtWAK1 free of AtGRP3 (*AtWAK1* overexpression or *grp3-1* plants), this signaling is impaired resulting in Al tolerance.

Interestingly, the levels of *AtWAK1* are induced by Al, while *AtGRP3* levels are not significantly induced (Fig 4C). This could be a strategy to accumulate AtWAK1 free of AtGRP3 that, according to Kohorn [47], would lead to more cell expansion. The first symptom of Al toxicity is the inhibition of root elongation, which occurs around 1–2 h after exposition to Al [50]. This fast response indicates that Al primarily inhibits cell elongation and expansion, although, in the long term, cell division is also affected [50, 51]. By increasing *AtWAK1* levels, the plant would enhance root elongation at least to a minimum, trying to overcome Al toxicity to some extent.

Our data indicates *AtGRP3* as a repressor of root growth during plant development and upon Al stress. Collectively, these results points for functional orthologues of *AtGRP3* as good targets for biotechnological approaches for Al tolerance, since knocking down these genes would not only lead to higher tolerance but also longer roots which could increases productivity.

## Supporting Information

S1 Fig *grp3-2* loss-of-function mutant analysis. **a** Relative expression of *AtGRP3* transcripts analyzed through real-time quantitative PCR of Col and *grp3-2* mutant. **b** Summarized data for root length measurements of 1-week-old plants. *Error bars* indicate standard error. * indicates p≤ 0.05 and *** indicates p≤ 0.005.
(TIF)

S1 Table List of qPCR primers. (DOCX)

## Abbreviations

GRP: glycine-rich protein
Al: Aluminum
RLK: receptor-like kinase
GFP: green fluorescence protein
KAPP: kinase-associated protein phosphatase
