# Supplementary figures and images for: AtGRP3 Is Implicated in Root Size and Aluminum Response Pathways in Arabidopsis
Source: PLoS One. 2016 Mar 3;11(3):e0150583. doi: 10.1371/journal.pone.0150583 (PMC4777284; doi:10.1371/journal.pone.0150583)

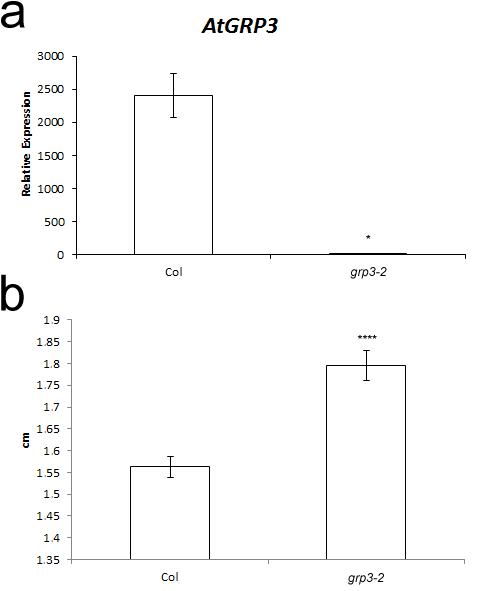

Supplement: S1 Fig — a Relative expression of AtGRP3 transcripts analyzed through real-time quantitative PCR of Col and grp3-2 mutant. b Summarized data for root length measurements of 1-week-old plants. Error bars indicate standard error. * indicates p≤ 0.05 and *** indicates p≤ 0.005. (TIF) [file pone.0150583.s001.tif]
