# Supplementary material for: AtGRP3 Is Implicated in Root Size and Aluminum Response Pathways in Arabidopsis
Source: PLoS One. 2016 Mar 3;11(3):e0150583. doi: 10.1371/journal.pone.0150583 (PMC4777284; doi:10.1371/journal.pone.0150583)
Supplement: S1 Table — (DOCX) [file pone.0150583.s002.docx]

| *At Code* | Primer name | | Primer sequence | | [mM] | Reference |  |
| --- | --- | --- | --- | --- | --- | --- | --- |
| At4g34270 | | TIP41L-RT-F  TIP41L-RT-R | | 5’-TTGGCTGAGAGTTGATGGTG-3’  5’-GGTCGCTCCAGACTGCTAAG-3’ | 300 | Alves-Ferreira, personal communication | |
| At5g43940 | | FDH1  FDH2 | | 5’-TGGGAAACCCATTTATCACTTCATG-3’  5’-CAGCAAGTCCAACAGTGCCAAG-3’ | 300 | [52] | |
| At4g39400 | | BRI1-RT-F  BRI1-RT-R | | 5’-AGCGATTAGCGGAAACAAAA-3’  5’-AGATGTTGCAGAGCAGAGCA-3’ | 300 | this paper | |
| At3g09840 | | CDC48-RT-F  CDC48-RT-R | | 5’-GTATGCGCGTAGGAGTGTGA-3’  5’-ACCGAACCCTCTAGACTGCT-3’ | 600 | this paper | |
| At5g64740 | | CESA6-RT-F  CESA6-RT-R | | 5’-AAGTGACGGTGTTCGTGATG-3’  5’-TCAAATCCGGGTCTCTTCTC-3’ | 300 | this paper | |
| At5g60920 | | COB-RT-F  COB-RT-R | | 5’-GGAACAAGCTTCGTCTCTGG-3’  5’-ATTTGGAGACGATGGAGGTG-3’ | 300 | this paper | |
| At5g06150 | | CYCB1;2-RT-F  CYCB1;2-RT-R | | 5’-TACTCGAAGTTTCCGTGCCCAGTT-3’  5’-TCTTTGGACCAAGAGCTGGAACCT-3’ | 300 | [53] | |
| At4g35620 | | CYCB2;2-RT-F  CYCB2;2-RT-R | | 5’-AGAGGTTCCTCAAGGCAGCTCAAT-3’  5’-GTGCTGTTCCATTCACTGAAGCCA-3’ | 600 | [53] | |
| At4g34160 | | CYCD3;1-RT-F  CYCD3;1-RT-F | | 5’-TGGGCAATCGTAGCCACTCCATAA-3’  5’-CACGTAAGGGCATTCGCGACATTT-3’ | 600 | [53] | |
| At1g12840 | | DET3-RT-F  DET3-RT-R | | 5’-GGCTGGTTTAGCTGGTGACT-3’  5’-GGAGAAGTGGCATCAAAAGC-3’ | 600 | this paper | |
| At3g19820 | | DWF1-RT-F  DWF1-RT-R | | 5’-TGACTGCTAGAGTGGAGCCT-3’  5’-AGCAACAACAGCGAGAGACA-3’ | 600 | this paper | |
| At2g06850 | | EXGT-A1-RT-F  EXGT-A1-RT-R | | 5’-AAGCTTCTGTGGAAGCCAAG-3’  5’-TTGTTCAGCGTCAAGGTCAC-3’ | 600 | this paper | |
| At2g37640 | | EXPA3-RT-F  EXPA3-RT-R | | 5’-CAGGGTACCTTGTCGGAAGA-3’  5’-AGTGATTGGCCGATGAGAAC-3’ | 300 | [54] | |
| At2g05520 | | GRP3-RT-F  GRP3-RT-R | | 5´-CTTCCAAGGCTTTGGTTCTG-3´  5´-AGCCACGTTGATCAGGTTTC-3’ | 600 | this paper | |
| At2g23430 | | ICK1-RT-F  ICK1-RT-R | | 5’-AGCTACGGAGCCGGAGAATTGTTT-3’  5’-GTCGACGTTTCAGTGTCACCATCT-3’ | 300 | [53] | |
| At5g49720 | | KOR1-RT-F  KOR1-RT-R | | 5’-GTCCAACGGAGCAGAAGAAG-3’  5’-CGGCAACAAGAGTACCAACA-3’ | 100 | this paper | |
| At1g05850 | | POM1-RT-F  POM1-RT-R | | 5’-TGCCCTCTTTGAGCCTCTTG-3’  5’-GCTGGCTACATGACCGAGAA-3’ | 100 | this paper | |
| At3g16640 | | TCTP-RT-F  TCTP-RT-R | | 5’-TCGTTGGGTTACTGTGGTCG-3’  5’-ATAGGGGACGACGACGAAAC-3’ | 300 | this paper | |
| At1g21250 | | WAK1-RT-F  WAK1-RT-R | | 5´-CGGATCCCAAAACCTGTAGA-3´  5´-GTATACAGGCAACGCCAAG-3’ | 600 | this paper | |

**References**

52. Braz ASK, Finnegan J, Waterhouse P, Margis R. A plant orthologue of RNase L Inhibitor (RLI) is induced in plants showing RNA interference. Mol Evol. 2004; 59: 20–30.

53. Li X, Yu E, Fan C, Zhang C, Fu T, Zhou Y. Developmental, cytological and transcriptional analysis of autotetraploid *Arabidopsis*. Planta. 2012; 236: 579-596.

54. Son S-H, Chang SC, Park CH, Kima S-K. Ethylene negatively regulates EXPA5 expression in *Arabidopsis thaliana*. Physiol Plant. 2012; 144: 254–262.
